# A Brain-Inspired Decision-Making Spiking Neural Network and Its Application in Unmanned Aerial Vehicle

**DOI:** 10.3389/fnbot.2018.00056

**Published:** 2018-09-11

**Authors:** Feifei Zhao, Yi Zeng, Bo Xu

**Affiliations:** ^1^Research Center for Brain-Inspired Intelligence, Institute of Automation, Chinese Academy of Sciences, Beijing, China; ^2^University of Chinese Academy of Sciences, Beijing, China; ^3^National Laboratory of Pattern Recognition, Institute of Automation, Chinese Academy of Sciences, Beijing, China; ^4^Center for Excellence in Brain Science and Intelligence Technology, Chinese Academy of Sciences, Shanghai, China

**Keywords:** spiking neural network, brain-inspired decision-making, dopamine regulation, multiple brain areas coordination, reinforcement learning, UAV autonomous learning

## Abstract

Decision-making is a crucial cognitive function for various animal species surviving in nature, and it is also a fundamental ability for intelligent agents. To make a step forward in the understanding of the computational mechanism of human-like decision-making, this paper proposes a brain-inspired decision-making spiking neural network (BDM-SNN) and applies it to decision-making tasks on intelligent agents. This paper makes the following contributions: (1) A spiking neural network (SNN) is used to model human decision-making neural circuit from both connectome and functional perspectives. (2) The proposed model combines dopamine and spike-timing-dependent plasticity (STDP) mechanisms to modulate the network learning process, which indicates more biological inspiration. (3) The model considers the effects of interactions among sub-areas in PFC on accelerating the learning process. (4) The proposed model can be easily applied to decision-making tasks in intelligent agents, such as an unmanned aerial vehicle (UAV) flying through a window and a UAV avoiding an obstacle. The experimental results support the effectiveness of the model. Compared with traditional reinforcement learning and existing biologically inspired methods, our method contains more biologically-inspired mechanistic principles, has greater accuracy and is faster.

## 1. Introduction

Brain-inspired neural networks investigate on the nature of intelligence from computational perspective and provide new opportunities to achieve the goal of human-like intelligence. The motivation of this paper is to build a brain-inspired cognitive computational model based on brain connectome and decision-making mechanism and apply it to decision-making tasks for intelligent agents.

According to recent advancement of Neuroscience research, multiple brain areas are involved and they coordinate with each other to realize brain decision-making. Every brain area plays a unique role in decision-making, and each of them complement with each other for accomplishing a decision-making task. The basal ganglia (BG) plays a central role in action selection and reinforcement learning (Mink and Thach, 1993; Mink, 1996; Redgrave et al., 1999). The BG contains a set of subcortical nuclei located in the midbrain, around the thalamus. The major nuclei of BG contains striatum, internal globus pallidus (GPi), subthalamic nucleus (STN), external globus pallidus (GPe), substantia nigra pars reticulata (SNr), substantia nigra pars compacta (SNc) and ventral tegmental area (VTA) (Alexander and Crutcher, 1990; Lanciego et al., 2012).

A large number of computational models of information processing in the BG have been developed in recent years. Kenji Doya et al. investigated detailed biological mechanisms of reinforcement learning and related computational modeling (Daw and Doya, 2006; Doya, 2007). Joel et al. showed the similarity between ventral striatum and critic function, as well as the similarity between dorsal striatum and actor function (Joel et al., 2002). To describe the real-world environment, a continuous time Actor-Critic model has been proposed. This method simulated the continuous temporal difference (TD) learning by using spiking neurons (Frémaux et al., 2013). However, the gradient descent method they used to update weights in learning process is different from the biological brain learning mechanism.

Current experimental evidence indicates that the decision-making mechanism contains direct pathway, indirect pathway and hyperdirect pathway. The detailed operational mechanism of BG is currently believed to be as follows. Activity in the direct pathway sends a “Go” signal to facilitate the response to a specific action, whereas activity in the indirect pathway sends a “No Go” signal to suppress a specific action. Striatum contains two subclasses cells: “Go” cell and “No Go” cell. The “Go” cell directly inhibits GPi, and has the disinhibition effect on thalamus, thus facilitating the response to a specific action. The “No Go” cell on the indirect pathway firstly inhibits GPe, then GPe inhibits GPi. Thus, “No Go” cell has the opposing effect on GPi, suppressing the response to the action in thalamus. The hyperdirect pathway from STN directly excites GPi (Alexander et al., 1986; Alexander and Crutcher, 1990; Percheron and Filion, 1991).

The dopamine (DA) from SNc/VTA modulates the activity of direct and indirect pathways by activating different receptors. The “Go” cell expresses the D1 receptor, and we call it StrD1. The “No Go” cell expresses the D2 receptor, and we call it StrD2. The DA regulation plays an important role in decision-making. When the executed action is correct, the increase in DA lead to enhancing the activity in direct pathway, and simultaneously suppressing indirect pathway. When the executed action is incorrect, depletion of DA has the opposite effect, enhancing the indirect pathway and suppressing the direct pathway (Geffen, 2000; Silkis, 2000). Frank et al. modeled the direct pathway and indirect pathway in brain decision-making with DA regulation in their model (Frank, 2005). This work only focused on the interactions among brain areas in BG, while lack of considerations on other associated important brain areas, such as STN on the hyperdirect pathway and cortical areas.

Inspirations only from basal ganglia system may not be enough, since coordinations with wider areas of the cortex (Alexander et al., 1986) and thalamus are missing (Silkis, 2000; Utter and Basso, 2008). In addition, with the current efforts, generation of appropriate action selection may be too slow to be applied to complex decision making tasks in natural scene for intelligent systems. Frank et al. considered the bias top-down control from orbitofrontal cortex (OFC) to BG. OFC represents both positive and negative reward by two separate sub-areas, medial OFC (MOFC) and lateral OFC (LOFC) (Elliott et al., 2000; O'Doherty et al., 2001). OFC also has a bias effect on BG by maintaining contextual reward in working memory (Tremblay and Schultz, 1999). Inspired by this mechanism, a relative reward method has been proposed in Zhao et al. (2017). However, these works are just the mathematical computational model without the support of biological realistic spiking neurons and spiking neural networks (SNN).

Although SNN has been adopted for modeling decision-making circuit in recent years (Stewart et al., 2010; Gurney et al., 2015), they are generally considered to get inspirations from the brain at relatively coarser scales. Stewart et al. (2010) used SNN to simulate the BG decision-making circuit, while it did not take Spike-timing-dependent plasticity (STDP) mechanism and the function of OFC into consideration. Gurney et al. proposed an SNN model with STDP mechanism to simulate the BG decision-making circuit (Gurney et al., 2015). However, this work also did not consider the effect of OFC.

In this paper, we propose a brain-inspired decision-making spiking neural network (BDM-SNN) model with a focus on the inspirations of brain decision-making circuits and mechanisms. This paper makes the following contributions: (1) We use SNN to model human decision-making neural circuit and mechanism. (2) We combine DA regulation with STDP mechanism to modulate the learning process of the network. (3) We consider the effect of OFC on the representation of positive and negative feedback. (4) We apply the proposed model to the unmanned aerial vehicles (UAV) autonomous decision-making tasks, including the UAV flying through a window task and the UAV obstacle avoidance task.

## 2. Materials and methods

### 2.1. The neuroanatomy of brain decision-making circuit

**Figure 1:**
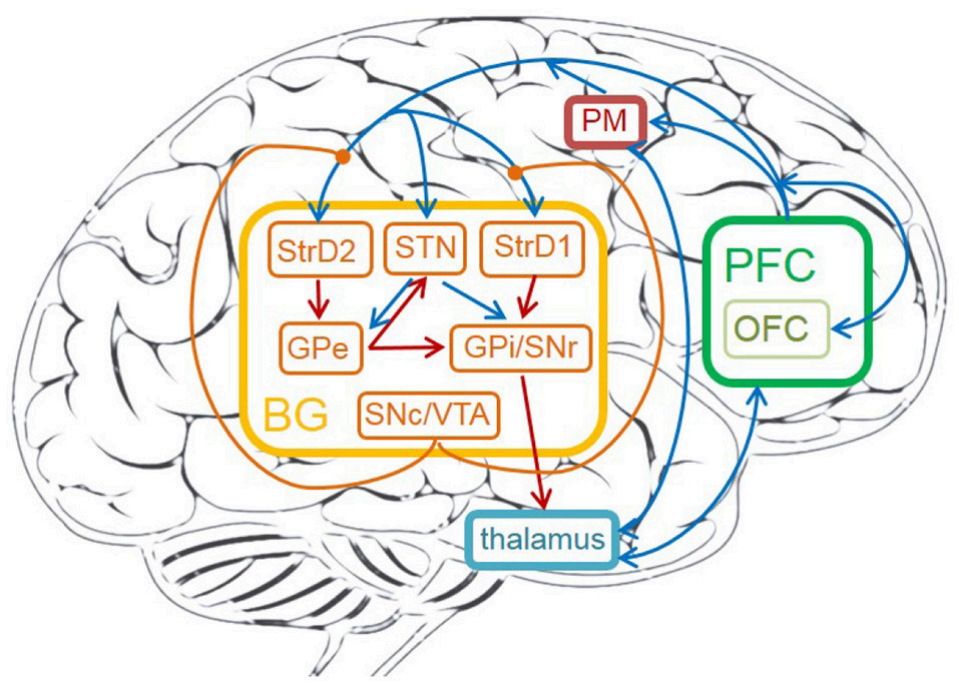
The cortico-basal ganglia-thalamo-cortical loop in human brain. The brain areas on the circuit contain: prefrontal cortex (PFC), OFC, premotor cortex (PM), thalamus, striatum (StrD1 and StrD2), GPi, STN, GPe, SNr, SNc, VTA. The orange brain areas represent sub-areas of BG. The blue lines represent excitatory connections, the red ones represent inhibitory connections, the orange ones represent modulatory connections.

The detailed cortico-basal ganglia-thalamo-cortical loop is depicted in Figure 1. The acronyms and full names of brain areas on the cortico-basal ganglia-thalamo-cortical loop are listed in Table 1. Here, prefrontal cortex (PFC) is the input of BG, and the BG projects to thalamus, then thalamus outputs action to premotor cortex (PM). This circuit includes direct, indirect and hyperdirect pathways of the BG. The direct pathway is: PFC excites StrD1, then StrD1 directly inhibits GPi (PFC-StrD1-GPi). The indirect pathway is: PFC excites StrD2, then StrD2 has a disinhibition effect on GPi through the inhibitory intermediate, GPe. The hyperdirect pathway is: PFC excites STN, then STN excites GPe and GPi. Direct pathway, indirect pathway and hyperdirect pathway are converged into GPi to output an inhibitory effect on thalamus. Thalamus outputs excitatory bias response to PM after combining the excitatory input from PFC and the inhibitory input from GPi (Albin et al., 1989; Silkis, 2000; Lanciego et al., 2012).

**Table 1:** The acronyms and full names of brain areas on the cortico-basal ganglia-thalamo-cortical loop.

**Acronyms**	**Full name**
BG	basal ganglia
GPi	internal globus pallidus
STN	subthalamic nucleus
GPe	external globus pallidus
SNr	substantia nigra pars reticulata
SNc	substantia nigra pars compacta
VTA	ventral tegmental area
DA	dopamine
PFC	prefrontal cortex
OFC	orbitofrontal cortex
MOFC	medial orbitofrontal cortex
LOFC	lateral orbitofrontal cortex
DLPFC	dorsolateral prefrontal cortex
PM	premotor cortex

DA plays an important role in the learning process of decision-making. The detailed learning mechanism is as the following: low and high DA respectively promote long-term depression (LTD) and long-term potentiation (LTP) on cortico-striatal synapses (Kerr and Wickens, 2001). Increased levels of DA promote LTP on StrD1 cells and LTD on StrD2 cells. Decreased levels of DA promote LTP on StrD2 cells and LTD on StrD1 cells (Shen et al., 2008). Direct pathway inhibits GPi, and thus has a disinhibition effect on thalamus, then sends a “Go” signal to PM. Indirect pathway disinhibits GPi, and thus has an inhibitory effect on thalamus, then sends a “No Go” signal to PM. Thus, increases in DA during positive feedback lead to reinforcing the selected response by facilitating the activity of StrD1 and suppressing the activity of StrD2. On the contrary, decreases in DA result in facilitating the activity of StrD2 and suppressing the activity of StrD1. By this way, the tendency of choosing this action will be weakened. To sum up, direct pathway sends a “Go” signal to facilitate a given response. Indirect pathway, with opposite effect on the thalamus, sends a “No Go” signal to suppress the response (Shen et al., 2008; Freeze et al., 2013). DA regulation can effectively enlarge the difference between two competitive direct pathway and indirect pathway, and is helpful for clear action selection.

The connections and functions of different brain areas for decision-making are as follows:

- **PFC**. PFC is important for quick decision-making. Firstly, PFC, which represents the environment information, is the input of BG. PFC also provides excitatory inputs to thalamus and PM (Rose and Woolsey, 1948). Secondly, PFC is considered to maintain contextual reward information in working memory, and it has a top-down bias effect on behavior selection process in BG (Riceberg and Shapiro, 2012). Thirdly, the sub-area of PFC, OFC, represents reinforcement values. The OFC represents both positive and negative reward in two separate sub-areas: MOFC and LOFC. Studies in O'Doherty et al. (2001) and Kringelbach (2005) showed that the MOFC tends to respond to positive reward of reinforcement values, whereas the LOFC is more active when representing negative rewards. Fourthly, dorsolateral prefrontal cortex (DLPFC) of PFC is responsible for representing state information (Barbey et al., 2013).
- **Striatum**. Striatum receives direct input from cortical areas such as PFC and PM. DA regulation focuses on the connections between PFC and striatum. The striatum has two types of DA receptors, D1 and D2. The StrD1 cells enhance the response of inputs, while the StrD2 cells have the contrary effect (Geffen, 2000). StrD1 and StrD2 are related to direct pathway and indirect pathway, respectively. The StrD1 projects directly to the GPi, and the StrD2 indirectly projects to GPi through the intermediate, GPe (Alexander et al., 1986; Alexander and Crutcher, 1990).
- **STN**. STN is the only area that elicits excitatory glutamatergic neurotransmitter in BG. STN receives excitatory input from PFC, and has excitatory connections with GPe and GPi. It also receives the inhibitory projection from GPe (Plenz and Kital, 1999). The time difference between direct and indirect pathway affects the decision-making process. The hyperdirect pathway from PFC to STN, then to GPe and GPi helps this process (Simon et al., 2013). STN plays important role in preventing making decision too fast.
- **GPe**. GPe receives inhibitory projection from the StrD2, and excitatory projection from STN. It has inhibitory connections with STN and GPi (Redgrave et al., 2010). GPe is the intermediate on the indirect pathway.
- **GPi/SNr**. GPi/SNr is the output nuclei of BG. It receives inhibitory input from StrD1, GPe, and excitatory input from STN. GPi provides inhibitory output to thalamus after combining the signals from direct pathway, indirect pathway and hyperdirect pathway (Redgrave et al., 2010).
- **SNc/VTA**. SNc/VTA is useful to elicit DA. Experiments show that DA could perform different functions, such as saliency sensitivity, reward sensitivity and punishment sensitivity (Schultz et al., 1993; Ethan et al., 2010). Due to the connections from SNc/VTA to OFC (Haber and Knutson, 2010), reward-related DA activates MOFC and punishment-related DA activates LOFC. DA is also used to modulate the connection weights between DLPFC and striatum (Nishi et al., 2011).
- **Thalamus**. Thalamus receives the inhibitory projection from GPi/SNr and excitatory input from PFC. It projects to PM after combining the BG signal and PFC signal (Silkis, 2000).
- **PM**. PM receives excitatory input from PFC and thalamus. It is useful to execute behaviors and provide feedbacks on the behavior information to striatum.

### 2.2. Network architecture

**Figure 2:**
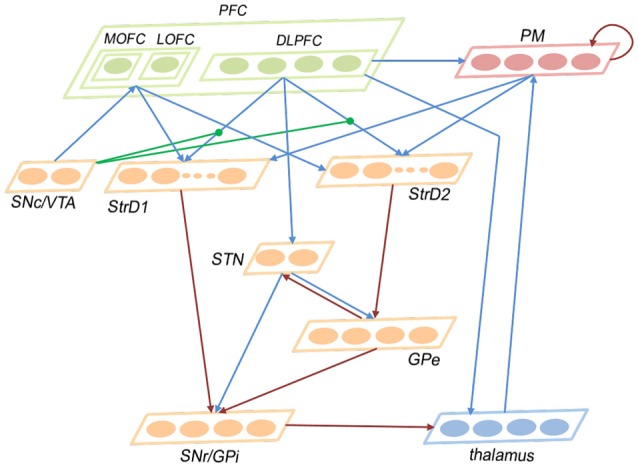
The network architecture of the BDM-SNN model. The orange brain areas represent sub-areas of BG. The green brain areas represent sub-areas of PFC. The blue lines represent excitatory connections, while the red ones represent inhibitory connections. The green connections represent DA modulatory connections.

This subsection introduces the architecture of the brain-inspired decision-making spiking neural network (BDM-SNN) model. Inspired by the decision-making circuits in human brain, our method simulates the connections and functions among these brain areas. The network architecture is depicted in Figure 2. The BDM-SNN model contains 11 modules which are corresponding to the key brain areas on the cortico-basal ganglia-thalamo-cortical loop. The modules of BDM-SNN model contain MOFC, LOFC, DLPFC, PM, SNc/VTA, StrD1, StrD2, STN, GPe, SNr/GPi, and thalamus. The StrD1 and StrD2 are corresponding to the “Go” and “No Go” cells in striatum. Other modules are corresponding to the functions of their brain area on cortico-basal ganglia-thalamo-cortical loop. The excitatory and inhibitory connections among different modules are inspired by the connections on the cortico-basal ganglia-thalamo-cortical loop. The DA regulates the connections between DLPFC and striatum, as shown as the green DA modulatory connections in Figure 2.

In this study, the numbers of neurons in different brain areas are defined according to their functions. For a decision-making task, we should predefine the possible state and action space. Suppose the number of state is *N*_*s*_, and the number of action is *N*_*a*_. The state information as the input is first transmitted to DLPFC, thus the number of neurons in DLPFC is *N*_*s*_. PM is responsible for executing action, thus it has *N*_*a*_ neurons. The state and action information from DLPFC and PM is transmitted to striatum (StrD1 and StrD2), thus the numbers of neurons in StrD1 and StrD2 are equal to *N*_*s*_ * *N*_*a*_. The GPi is the output area of BG and is responsible for action selection, thus the number of neurons in GPi is *N*_*a*_. Thalamus receives the projection from GPi and transmits action to PM, thus the number of neurons in thalamus is *N*_*a*_. GPe is the intermediate on the indirect pathway, thus the number of neurons in GPe is *N*_*a*_. MOFC responds to the positive feedback, and LOFC responds to negative feedback, thus the numbers of neurons in MOFC and LOFC are 1, respectively. SNc/VTA is the input of MOFC and LOFC, thus we assign two neurons for SNc/VTA with one related to positive feedback and another one related to negative feedback. The STN is the brain area on hyperdirect pathway, and we assign two neurons for it. The number of neurons in different brain areas are listed in Table 2.

**Table 2:** The number of neurons in different brain areas.

**Brain areas**	**DLPFC**	**PM**	**Thalamus**	**StrD1**	**StrD2**	**Gpi**	**Gpe**	**STN**	**VTA**	**MOFC**	**LOFC**
number	*N*_*s*_	*N*_*a*_	*N*_*a*_	*N*_s_*N_*a*_	*N*_s_*N_*a*_	*N*_*a*_	*N*_*a*_	2	2	1	1

*N_s_ represents the number of state space, and N_a_ represents the number of action space*.

The ways of connections among different areas are based on their functions and are listed in Table 3. Here, full connection means all-to-all connection. Specific connection means the connection between specific state or action and specific state-action pair. The state and executed action from DLPFC and PM are transmitted to StrD1 and StrD2. StrD1, and StrD2 display all the state-action pairs. As a result, specific connection means the connection from specific state (DLPFC) and action (PM) to state-action pair (StrD1 and StrD2). Two neurons in SNc/VTA are corresponding to positive and negative feedback, respectively. The positive one (reward-related DA) is connected to MOFC, and the negative one (punishment-related DA) is connected to LOFC. Here, the PM selects action on the basis of a competitive winner-takes-all (WTA) process. This is implemented via lateral inhibition among PM neurons.

**Table 3:** The type of connections among different areas.

**Connection**	**The type of connection**
DLPFC-PM	full connection
DLPFC-StrD1	specific connection
DLPFC-StrD2	specific connection
PM-StrD1	specific connection
PM-StrD2	specific connection
StrD1-SNr/GPi	specific connection
StrD2-Gpe	specific connection
Gpe-SNr/Gpi	one-to-one connection
DLPFC-STN	full connection
STN-Gpe	full connection
STN-SNr/Gpi	full connection
Gpe-STN	full connection
SNc/VTA-MOFC	one neuron connects to MOFC
SNc/VTA-LOFC	the other one neuron connects to LOFC
MOFC-StrD1	full connection
MOFC-StrD2	full connection
LOFC-StrD1	full connection
LOFC-StrD2	full connection
SNr/Gpi-thalamus	one-to-one connection
DLPFC-thalamus	full connection
thalamus-PM	one-to-one connection
PM-PM	lateral inhibition

*Here, full connection means all-to-all connection. Specific connection means the connection between specific state or action and specific state-action pair*.

### 2.3. Network implementation

This subsection introduces the concrete design and implementation of the BDM-SNN model. SNN is considered as the third generation of Artificial Neural Networks (Maass, 1997). It encodes the information in spike trains instead of spike rates as in the conventional Artificial Neural Networks (Hopfield, 1995). SNN is highly inspired by the synaptic interactions between neurons in the brain, and it takes into account the time factor of spike firing. The biological neuron model and the synaptic plasticity model are more biologically plausible, and more computationally powerful than other alternative networks (Maass, 1999; Bohte, 2004; Paugam-Moisy and Bohte, 2012). In this paper, we use SNN to model brain decision-making circuit. In this model, every neuron in DLPFC represents one state. The visual input is firstly preprocessed and assigned to a state. Then the corresponding neuron in DLPFC receives a constant input to excite this neuron. The action generated by this model is based on the first spiking neuron in PM area. Every action is corresponding to a neuron in PM. Then the first spiking action wins the competition and is executed. Delay coding method is used in this model where a stronger stimulus makes neurons fire earlier than weaker ones. The neuron model and synaptic plasticity model are as follows.

**1. Neuron Model**. To make a balance on the biologically realistic consideration and computational efficiency, the Izhikevich neuron model is applied in our model to build the brain-inspired SNN. It has more ionic dynamics than leaky integrate-and-fire (LIF) (Abbott, 1999) model, and computationally effective than the HodgkinHuxley model (Hodgkin and Huxley, 1952). Izhikevich introduced a neuron model that is capable of producing many patterns of biological neurons, which is as biologically plausible as the Hodgkin-Huxley model, yet as computationally efficient as the integrate and-fire model (Izhikevich, 2003). The neuron model is shown in Equations 1–4, where *v* represents the membrane potential of the spiking neuron, and *u* represents a membrane recovery variable. *a*, *b*, *c*, *d* are parameters to control the type of spiking dynamics. *I* is input. Each neuron receives weighted input from presynaptic neuron as Equation 3 calculated. *w*_*ji*_ is the strength of the connection from the *jth* neuron to the *ith* neuron. *o*_*j*_ is the output of the presynaptic neuron: 1 if *v*_*j*_ ≥ 30*mV*, and 0 otherwise. When the membrane potential *v* exceeds its peak of 30 mV, an action potential (spike) occurs, and the membrane potential is reset to its initial value, *c*, and the recovery variable is incremented by *d*. Izhikevich neuron model could be mainly classified into two categories: (1) Excitatory neurons: Regular Spiking (RS), Intrinsically Bursting (IB) and Chattering (CH). (2) Inhibitory neuron: Low-Threshold Spiking (LTS) and Fast Spiking (FS). The different neuron models correspond to different values of the parameters *a*, *b*, *c*, *d*. In this paper, we use RS neuron because it “fire a few spikes with short interspike period and then the period increases” (Izhikevich, 2003). The parameters of RS are: *a* = 0.02, *b* = 0.2, *c* = −65, *d* = 8.

(1) $$\begin{array}{l} v^{\prime }=0.04v^{2}+5v+140-u+I \end{array}$$

(2) $$\begin{array}{l} u^{\prime }=a\left(bv-u\right) \end{array}$$

(3) $$\begin{array}{l} I_{i}=\overset{N}{\underset{j=1}{\sum }}w_{ji}o_{j} \end{array}$$

(4) $$\begin{array}{l} if\;v\geq 30mV,then\;\{\begin{array}{c} v\leftarrow c\\ u\leftarrow u+d \end{array} \end{array}$$

**2. Synaptic Plasticity**. Spike Timing Dependent Plasticity (STDP) is one of the most important learning principle for the biological brain. STDP postulates that the strength of the synapse is dependent on the spike timing difference of the pre- and post-neuron (Gerstner et al., 1996; Bell et al., 1997; Bi and Poo, 1998; Poo, 2008). Here we use STDP to learn synaptic weights according to the relative time between spikes of presynaptic and postsynaptic neurons. The modulation principle is that if the postsynaptic neuron fires a few milliseconds after the presynaptic neuron, connection between the neurons will be strengthened, otherwise, the connection will be weakened (Nishiyama et al., 2000; Wittenberg and Wang, 2006). The update function is shown in Equation 5, where *A*_+_ and *A*_−_ are learning rates. τ_−_ and τ_+_ are time constant, and Δ*t*_*i*_ is the delay time from the presynaptic spike to the postsynaptic spike. Here, *A*_+_ = 0.925, *A*_−_ = 0.9, τ_−_ = τ_+_ = 20.

(5) $$\begin{array}{l} \Delta w_{j}=\{\begin{array}{cc} A_{+}e^{\left(\Delta t_{i}/\tau_{+}\right)} & \Delta t_{i}<0\\ -A_{-}e^{\left(-\Delta t_{i}/\tau_{-}\right)} & \Delta t_{i}>0 \end{array} \end{array}$$

### 2.4. Decision making mechanisms

#### 2.4.1. Continuous DA regulation

The reward signal is encoded in the activity of midbrain DA neurons. With rewards, neurons in SNc/VTA generate bursting activities (Mirenowicz and Schultz, 1996; Schultz, 2000). In human brain, DA reward signals are continuously elicited at every moment. We use continuous reward function for every discrete state to emulate continuous DA signals at abstract level (Zhao et al., 2017). By this way, the reward is different at different moment even if in the same state. The reward function at time *t* is calculated by Equation 6. Here, we note that the *r*_*t*_ is not the final result of the actual reward, but an evaluation of current state at time *t*. The *Eva* (*s*_*t*_) is a continuous function which is changed along with time. For every state, *R*_*b*_ (*s*_*t*_) represents the basic reward value of current state. If the current state and the next state are the same, the *R*_*b*_ (*s*_*t*_) will not be changed. Then *Eva* (*s*_*t*_) is useful to deal with this condition, since it is changed at every moment.

(6) $$\begin{array}{l} r_{t}=R_{b}\left(s_{t}\right)+\alpha Eva\left(s_{t}\right) \end{array}$$

where *R*_*b*_ (*s*_*t*_) represents the basic reward value of current state, and *Eva* (*s*_*t*_) represents the evaluation reward of current moment, α is the scale factor.

**Figure 3:**
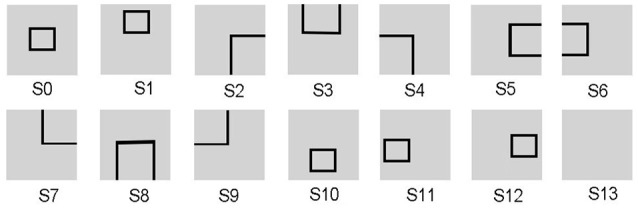
The states of the UAV flying through a window task. The visual input is firstly preprocessed and assigned to state. Then the corresponding neuron in DLPFC receives a constant input to excite this neuron.

Since the evaluation standards of different states are highly relevant to tasks, the reward function needs to be appropriately predefined for different tasks. For the UAV flying through a window task, the states are divided into 14 groups, as shown in Figure 3. The state division is based on the relative position between the UAV and the window. The aim of classifying states is to set some distinguishable states in advance, which contain all the conditions the UAV might observe. The classification results vary with each individual. Then all the 14 states are classified into four categories in order to design the continuous reward functions, respectively. The four categories contain: *C*_1_ = {*s*_13_}, *C*_2_ = {*s*_2_, *s*_3_, *s*_4_, *s*_5_, *s*_6_, *s*_7_, *s*_8_, *s*_9_}, *C*_3_ = {*s*_1_, *s*_10_, *s*_11_, *s*_12_}, *C*_4_ = {*s*_0_}. Every category has its own basic reward *R*_*b*_ and evaluation function *Eva*_*w*_ (*t*). Here, we give *R*_*b*_ (*C*_1_) = −1, 000, *R*_*b*_ (*C*_2_) = −600, *R*_*b*_ (*C*_3_) = −300, *R*_*b*_ (*C*_4_) = 1000. The continuous *Eva*_*w*_ (*t*) is the evaluation of current state at current time. *Eva*_*w*_ (*t*) keeps increasing when the UAV gets close to the center of the window. At time *t*, the *Eva*_*w*_ (*t*) is calculated by Equation 7.

(7) $$\begin{array}{l} Eva_{w}\left(t\right)=\{\begin{array}{cc} \frac{win_{w}+win_{h}}{I_{w}+I_{h}} & s_{t}\in C_{2}\\ \frac{-\left(\left|G_{u}-G_{d}|+|G_{l}-G_{r}\right|\right)}{I_{w}+I_{h}} & s_{t}\in C_{3}\\ 0 & C_{1}\\ 1,000 & C_{4} \end{array} \end{array}$$

where *I*_*w*_ is the width of the visual input of UAV, and *I*_*h*_ is the height of the visual input of UAV. *win*_*w*_ is the width of the window in the image, and *win*_*h*_ is the height of the window in the image. *G*_*u*_, *G*_*d*_, *G*_*l*_, *G*_*r*_ represent four distances (up, down, left, right) between the borders of the windows and the borders of the visual inputs, respectively.

#### 2.4.2. Working memory

In human brain, the PFC is involved in flexible and fast decision making through maintaining contextual reward information in working memory, and then uses this information to control the behavior selection in the next trial. After executing an action, reward information is rapidly encoded and maintained in working memory, and the actual expected reward is calculated by comparing with contextual reward in working memory (Tremblay and Schultz, 1999; Riceberg and Shapiro, 2012). Inspired by the expected reward estimation in PFC, the actual reward *r*_*end*_ is calculated by comparing the current reward *r*_*t*_ (which is maintained in working memory) with the next reward *r*_*t*+1_. Here, the actual reward *r*_*end*_ determines the value of executed action, and is useful for network learning.

(8) $$\begin{array}{l} r_{end}=r_{t+1}-r_{t} \end{array}$$

where *r*_*t*_ is the reward at time *t*, and *r*_*t* + 1_ is the reward at time *t* + 1. Here, *r*_*t*_ and *r*_*t* + 1_ represent the evaluation of visual inputs at time *t* and *t* + 1, which are not the actual reward used for learning.

#### 2.4.3. Combining DA regulation with STDP

Phasic activity of DA neurons signals prediction error, and is useful to dynamically modulate the behavior choice in BG (Schultz et al., 1997; Schultz, 1998). It is suggested that positive and negative feedback have opposing effects on DA release. Positive feedback leads to phasic bursts of DA, while negative feedback leads to phasic dips of DA (Schultz et al., 1993; Schultz, 1998). Increased levels of DA activate the direct pathway (synapses between DLPFC and StrD1) and suppress the indirect pathway (synapses between DLPFC and StrD2) (Geffen, 2000). Decreased levels of DA have the opposite effect, activate the indirect pathway and suppress the direct pathway. The DA driven plasticity involves the specific synapses between current state neuron of DLPFC and current state-executed action pair neuron in striatum.

In summary, after executing an action, the level of DA increases when the environment provides a positive reward feedback, and the level of DA decreases when environment provides a negative reward feedback. The increased levels of DA strengthen the weights between DLPFC and StrD1 and weaken the weights between DLPFC and StrD2. The decreased levels of DA strengthen the weights between DLPFC and StrD2 and weaken the weights between DLPFC and StrD1. In this paper, the actual reward *r*_*end*_ represents the feedback of environment (prediction error), and *r*_*end*_>0 represents the positive feedback while *r*_*end*_ ≤ 0 represents the negative feedback. Based on the DA regulation mechanism, we simplify the DA concentration by Equations 9, 10. The reason of having this kind of abstract calculation of DA concentration is that the uniform modulatory factor for synapses on direct and indirect pathways makes the network controllable and stable.

(9) $$\begin{array}{l} {DA}_{D1}=\{\begin{array}{cc} 2 & r_{end}>0\\ 0.5 & r_{end}\leq 0 \end{array} \end{array}$$

(10) $$\begin{array}{l} {DA}_{D2}=\{\begin{array}{cc} 0.5 & r_{end}>0\\ 2 & r_{end}\leq 0 \end{array} \end{array}$$

where *DA*_*D*1_ is the DA concentration on StrD1, and *DA*_*D*2_ is the DA concentration on StrD2.

Because DA is a type of neurotransmitter, it modulates the synaptic weights by Equations 11 and 12:

(11) $$\begin{array}{l} W_{DLPFC-StrD1}=W_{DLPFC-StrD1}\times {DA}_{D1} \end{array}$$

(12) $$\begin{array}{l} W_{DLPFC-StrD2}=W_{DLPFC-StrD2}\times {DA}_{D2} \end{array}$$

here, *W*_*DLPFC*−*StrD*1_ represents synaptic weights between DLPFC and StrD1, and *W*_*DLPFC*−*StrD*2_ represents synaptic weights between DLPFC and StrD2.

**Figure 4:**
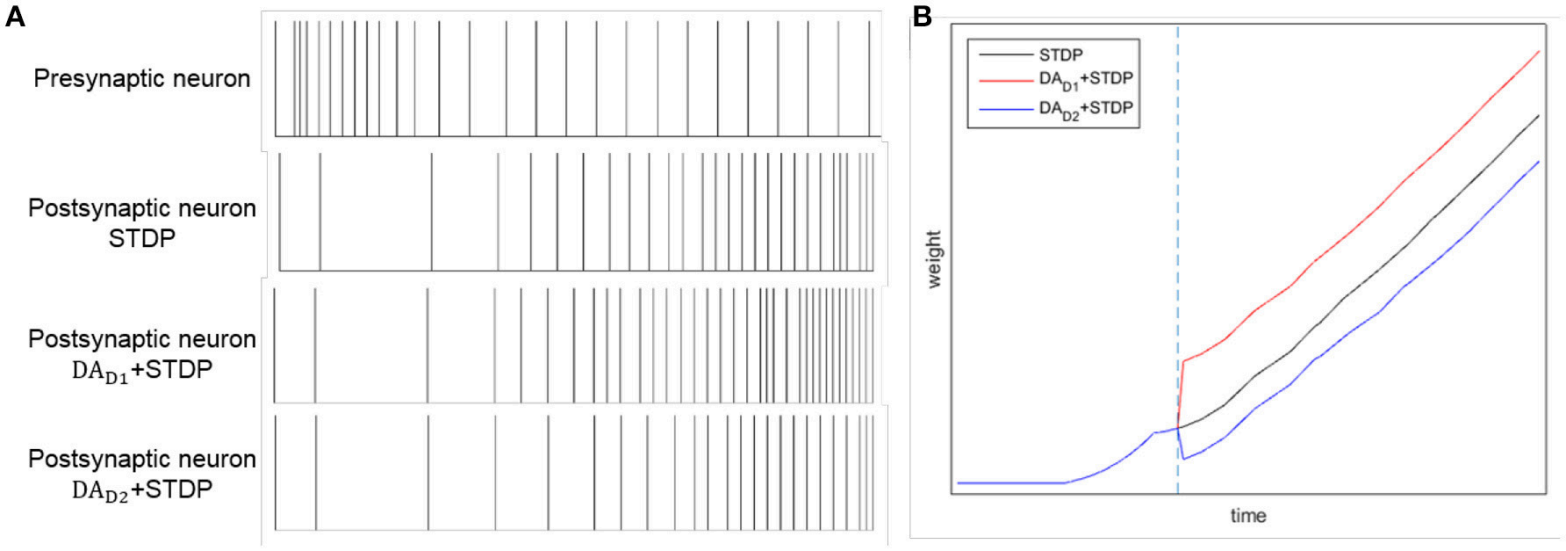
The effects of STDP, *DA*_*D*1_ + *STDP* and *DA*_*D*2_ + *STDP*. Considering a synaptic connection from one presynaptic neuron to one postsynaptic neuron, we provide a constant input to presynaptic neuron. And we analyze the spikes of the neurons with STDP, *DA*_*D*1_ + *STDP* and *DA*_*D*2_ + *STDP* regulation. **(A):** The spikes of presynaptic neuron and postsynaptic neuron by STDP, *DA*_*D*1_ + *STDP* and *DA*_*D*2_ + *STDP* regulation. **(B):** The change of weights between two neurons with STDP, *DA*_*D*1_ + *STDP* and *DA*_*D*2_ + *STDP* regulation. The blue dotted line represents the time of obtaining reward. Here, *DA*_*D*1_ + *STDP* represents the effect of StrD1 when obtaining reward, and *DA*_*D*2_ + *STDP* represents the effect of StrD2 when obtaining reward.

By combining DA regulation and STDP, the difference between two competing pathways (direct pathway and indirect pathway) will be enlarged. Here we test the comparative effects of only STDP and combining DA with STDP, as Figure 4 shown. Considering a synaptic connection from one presynaptic neuron to one postsynaptic neuron, we provide a constant input to presynaptic neuron. We test three conditions: only with STDP, increased level of DA for direct pathway (*DA*_*D*1_ regulates *W*_*DLPFC*−*StrD*1_), and increased level of DA for indirect pathway (*DA*_*D*2_ regulates *W*_*DLPFC*−*StrD*2_). The reward is provided at the time of blue dotted line in Figure 4B. The spikes of postsynaptic neuron with *DA*_*D*1_ + *STDP* are similar to the spikes of StrD1. The spikes of postsynaptic neuron with *DA*_*D*2_ + *STDP* are similar to the spikes of StrD2. Figure 4A indicates that the spikes of postsynaptic neuron with *DA*_*D*1_ + *STDP* are denser than only with STDP, and the spikes with *DA*_*D*2_ + *STDP* are sparser than only with STDP. Figure 4B shows that the weight of *DA*_*D*1_ + *STDP* is larger than only with STDP, and the weight of *DA*_*D*2_ + *STDP* is smaller than only with STDP. Thus, the combination of DA regulation and STDP could enlarge the difference between direct pathway and indirect pathway.

STDP and DA regulation collectively contribute to the learning process. The BDM-SNN model shows two effects by combining STDP and DA regulation:

**(1)** Increases in DA lead to the activity of StrD1 followed by the activity of DLPFC. Then, the weights between DLPFC and StrD1 will be strengthened when the environment provides positive reward feedback according to STDP mechanism. Besides, the weights between DLPFC and StrD2 will be strengthened when the environment provides negative reward feedback. As a result, the effects of STDP and DA regulation are coordinated to help quick decision-making.

**(2)** Although DA only acts on the connections between DLPFC and striatum, it influences the whole network by combining with STDP. When the level of DA increases, the activity of StrD1 is facilitated and the activity of StrD2 is suppressed. Due to the STDP mechanism, the connection weights between MOFC and StrD1 will be strengthened, and the connection weights between MOFC and StrD2 will be weakened. As for LOFC, the connection weights between LOFC and StrD2 will be strengthened, and the connection weights between LOFC and StrD1 will be weakened. By this way, when the environment provides positive reward, the StrD1 will be activated more quickly due to the input from MOFC. When the environment provides negative reward, the StrD2 will be activated more quickly due to the input from LOFC.

### 2.5. Experimental procedure

**Figure 5:**
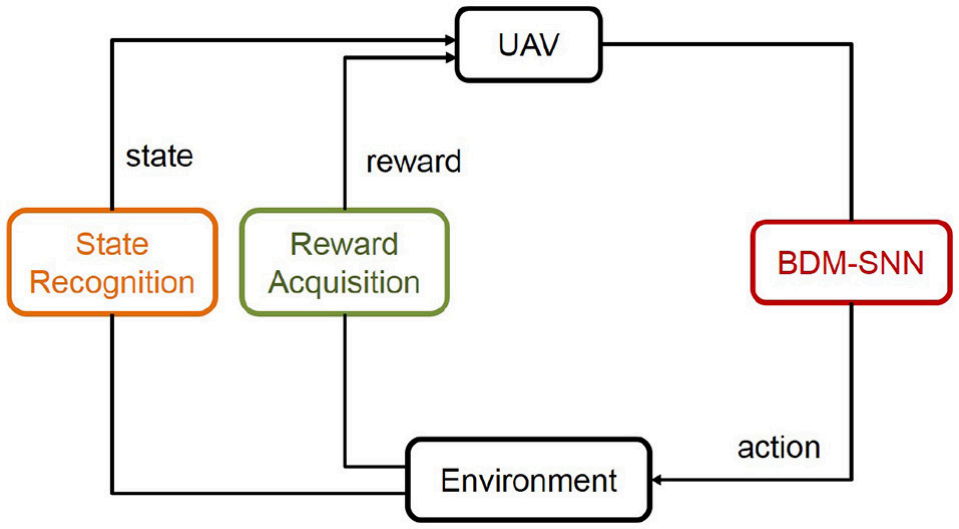
The basic procedure of the UAV decision-making. The state and reward information is transmitted to the BDM-SNN model, and the output of the model is the action. This figure shows the learning process, which will be circulated until the UAV reaches goal state.

This subsection introduces the application of the BDM-SNN model on the UAV decision-making tasks. All of the experiments are conducted on the DJI MATRICE 100 UAV. A 2.4 GHz wireless digital video camera (1/4 CCD) is used to acquire visual inputs. Here we test two decision making experiments: the UAV flying through a window task and the UAV obstacle avoidance task. The basic procedure of the UAV decision-making is depicted in Figure 5. Action selection is based on the output of BDM-SNN model. After the UAV executes an action, the environment produce feedback reward and state to the BDM-SNN model. State recognition module is used to classify states, and reward acquisition module is used to obtain rewards. This cycle will be circulated until the UAV reaches goal state and finishes the task. The visual input is first preprocessed and assigned to state. Then the corresponding neuron in DLPFC receives a constant input to excite this neuron. For the UAV flying through a window task, the state space and the reward function has been introduced in section Continuous DA Regulation. The action space contains four directions: ←, ↑, → , ↓, which means the UAV can fly toward left, up, right and down. For obstacle avoidance task, there are two states: obstacle situated in the left and the right part of the UAV's vision. The actions for the UAV obstacle avoidance task contain flying toward left and right. The basic reward *R*_*b*_ = –500. The evaluation function *Eva*_*d*_ (*t*) is shown in Equation 13.

(13) $$\begin{array}{l} Eva_{d}\left(t\right)=\max\left(width-x,x\right) \end{array}$$

where *width* represents the width of the visual input of the UAV, and *x* represents the x-coordinate of obstacle.

The detailed working procedure of the BDM-SNN model on the UAV decision-making is shown in Algorithm 1.

**Algorithm 1:** The working procedure of the BDM-SNN model for UAV decision-making.

**Require:** Initial state, initial time *t* = 1.
1: **repeat**
2: At state *s*_*t*_, calculating reward *r*_*t*_;
3: At state *s*_*t*_, the BDM-SNN model outputs action *a*_*t*_ at time *t*;
4: At state *s*_*t*+1_, calculating reward *r*_*t*+1_;
5: Calculating actual reward *r*_*end*_;
6: Calculating DA concentration *DA*_*D*1_ and *DA*_*D*2_;
7: Using DA concentration to update the connection weights *W*_*DLPFC*−*DtrD*1_ and *W*_*DLPFC*−*StrD*2_;
8: At state *s*_*t*+1_, the BDM-SNN model outputs action *a*_*t*+1_ at time *t* + 1;
9: *t* ← *t* + 1;
10: **until** The UAV finishes the task;

## 3. Results

### 3.1. The UAV flying through a window task

#### 3.1.1. Experimental results

**Figure 6:**
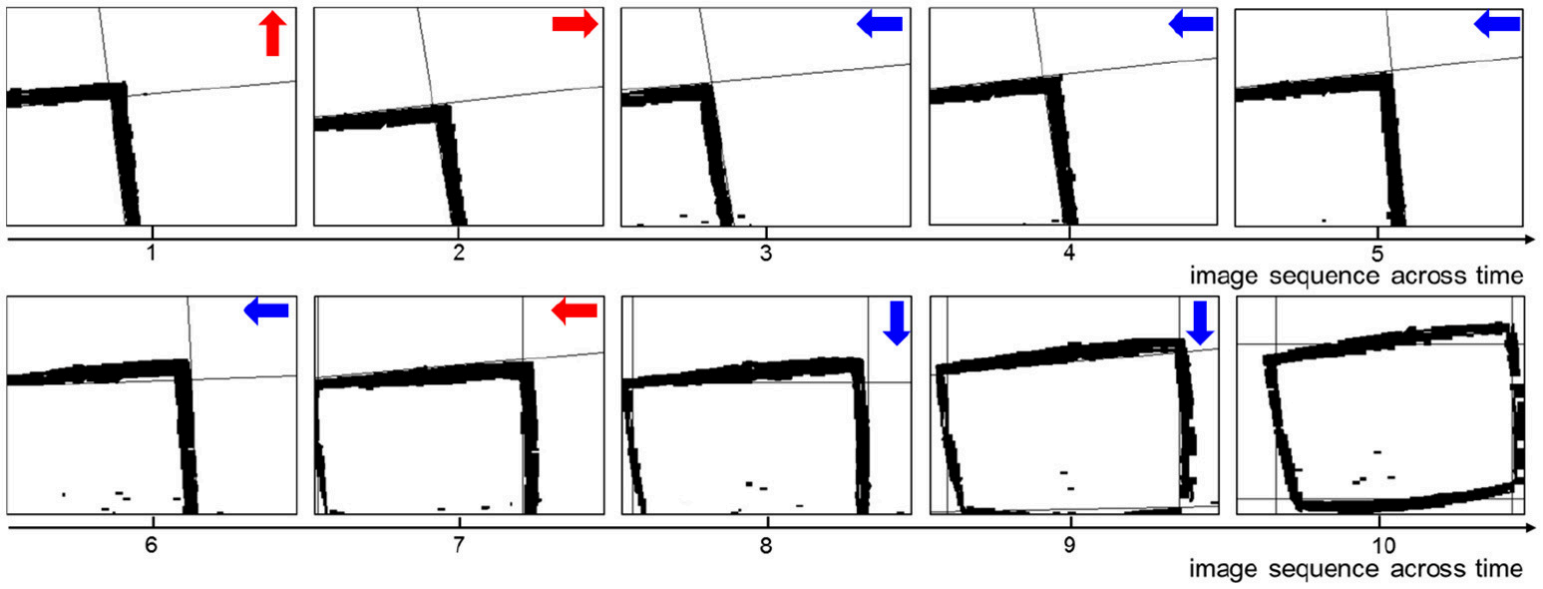
The image sequence during the UAV flying through window. The red arrows represent incorrect actions, and the blue arrows represent correct actions.

A key sequence of images during the decision making process is shown in Figure 6. It is obvious that the UAV modulates its movement toward the center of the window. When the UAV encounters a state for the first time, it probably chooses an incorrect action. Then the UAV optimizes the policy to weaken the tendency of selecting this action in order to select the correct action when encountering this state again. In every state, the UAV can learn the correct policy within three trials. As long as the UAV chooses the correct action, it will remember this action. On the contrary, if the UAV chooses the incorrect action, it will remember to avoid this action too.

#### 3.1.2. Model analysis

Although the experimental results on the UAV flying through a window task are similar to the result in Zhao et al. (2017), the methods they used are totally different. The method in Zhao et al. (2017) is mathematical optimization of Actor-Critic method. It is a brain inspired method by introducing the top-down bias effect of OFC. The method introduced in this paper uses the SNN to simulate the connections and functions of brain areas on decision-making circuit. We consider the collective contribution of three pathways (direct pathway, indirect pathway and hyperdirect pathway), which is more brain-like and biologically realistic. Now, we analyze the effectiveness of the BDM-SNN model.

**Figure 7:**
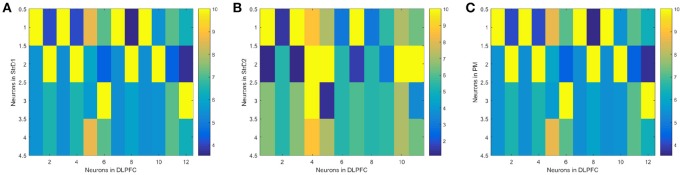
After the learning process, the weight distribution between DLPFC and StrD1 **(A)**, DLPFC and StrD2 **(B)**, DLPFC, and PM **(C)**. X axis represents the state, and y axis represents the action. The color in the rectangle of state-action pair represents the value of weight, and the closer the color is to yellow, the larger the weight is.

**Figure 8:**
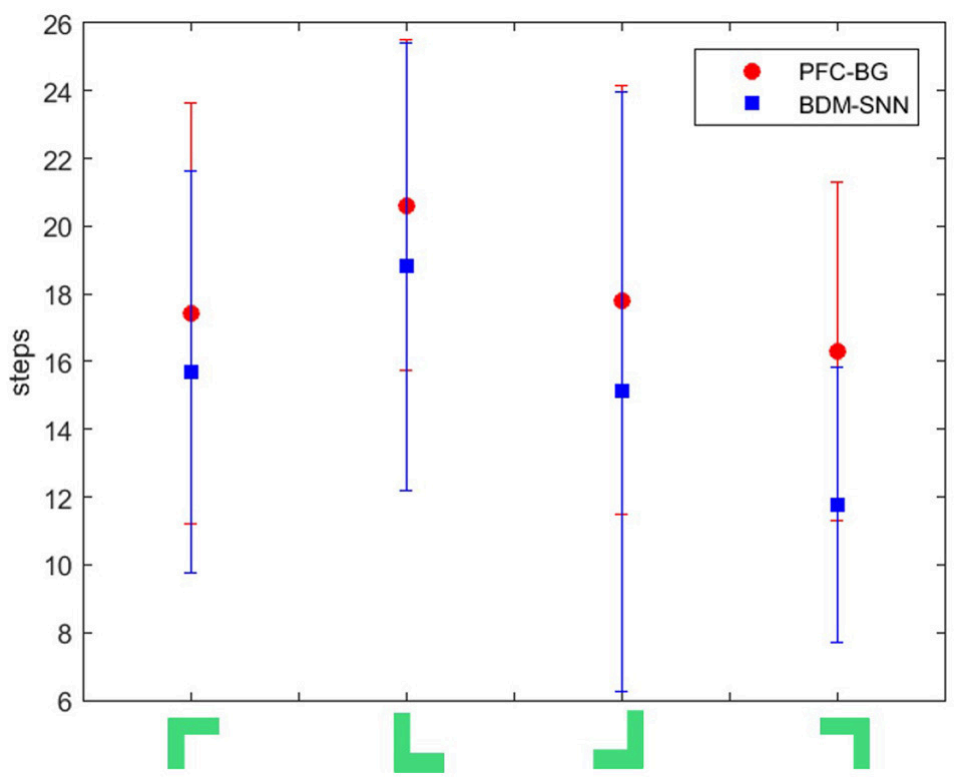
The required steps from different initial states to goal by the PFC-BG model and the BDM-SNN model. The X axis represents the four initial states. The red circles represent the average required steps of the PFC-BG model. The blue squares represent the average required steps of the BDM-SNN model. For each method, we conduct 15 experiments in every initial state.

(1) The effectiveness of the BDM-SNN model. The key learning process is the combination of DA regulation and STDP. DA regulation focuses on the connective weights between the DLPFC and the striatum. During the learning process, the weights of DLPFC-StrD1, DLPFC-StrD2, DLPFC-PM have been updated to learn the correct behaviors. After finishing the task, the weights between every state-action pair have been learned and optimized. Figure 7A shows the weights distribution between DLPFC and StrD1. Figure 7B shows the weights distribution between DLPFC and StrD2. Figure 7C shows the weights distribution between DLPFC and PM. The color in the rectangle of state-action pair represents the value of weight, and the closer the color is to yellow, the larger the weight is. When the UAV tries a correct action, the weights between DLPFC and StrD1 will be strengthened, while the weights between DLPFC and StrD2 will be weakened. By this way, the weights between DLPFC and PM will be strengthened to choose this action. When the UAV tries an incorrect action, the weights between DLPFC and StrD1 will be weakened, while the weights between DLPFC and StrD2 will be strengthened. Then the weights between DLPFC and PM will be weakened. The weights distributions in Figures 7A,C indicate that the weights between specific state and correct action are larger than the weights between specific state and incorrect action on the connections of DLPFC-StrD1 and DLPFC-PM. Figure 7B indicates that the weights between specific state and incorrect action are larger than the weights between specific state and correct action on the connections of DLPFC-StrD2. This is in line with prediction because the effect of DLPFC-StrD2 is inhibiting the action selection.

(2) Comparative evaluation. We compare the BDM-SNN model with the PFC-BG model with the same initial state (Zhao et al., 2017). The UAV is first situated in the upper-left, upper-right, lower-left and lower-right corners of window, and we conducted 15 experiments in every corner. The required steps from initial position to goal state (window's center) are recorded, and the average and variance are depicted in Figure 8. The results indicate that the BDM-SNN model needs fewer steps to finish the task compared to the PFC-BG model. The slight advantage is usually occurred when the UAV moves from one state to another state. The reason may be that the SNN model is dynamic and flexible to adapt new state while the mathematical optimization is relatively not that flexible. Because it has been indicated that Q-learning and Actor-Critic methods could not finish the UAV flying through a window task based on the discussion in Zhao et al. (2017), our method is superior to Q-learning and Actor-Critic methods.

### 3.2. The UAV obstacle avoidance task

#### 3.2.1. Experimental results

**Figure 9:**
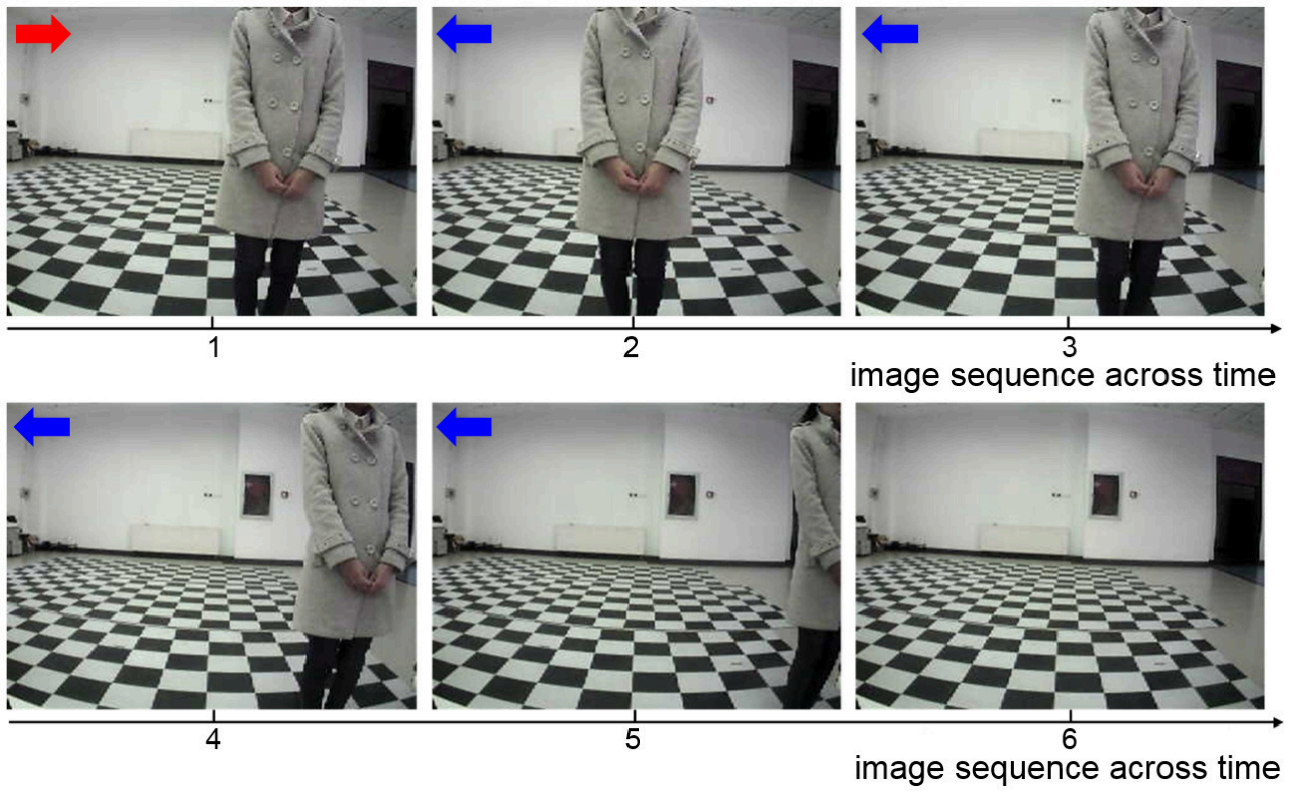
The sequence of images during the UAV obstacle avoidance process. The red arrow represents incorrect action, and the blue arrows represent correct actions.

The key sequence of images during the obstacle avoidance process is shown in Figure 9. Firstly, the obstacle is situated in the right of the UAV's vision. After moving a step, the obstacle is situated in the center of the UAV's vision. This is an incorrect action, and the UAV modifies the strategy and chooses leftwards action. After moving left, the obstacle is situated in the right of the UAV's vision. Then, the UAV learns this correct action and continues moving left to avoid the obstacle.

#### 3.2.2. Model analysis

**Figure 10:**
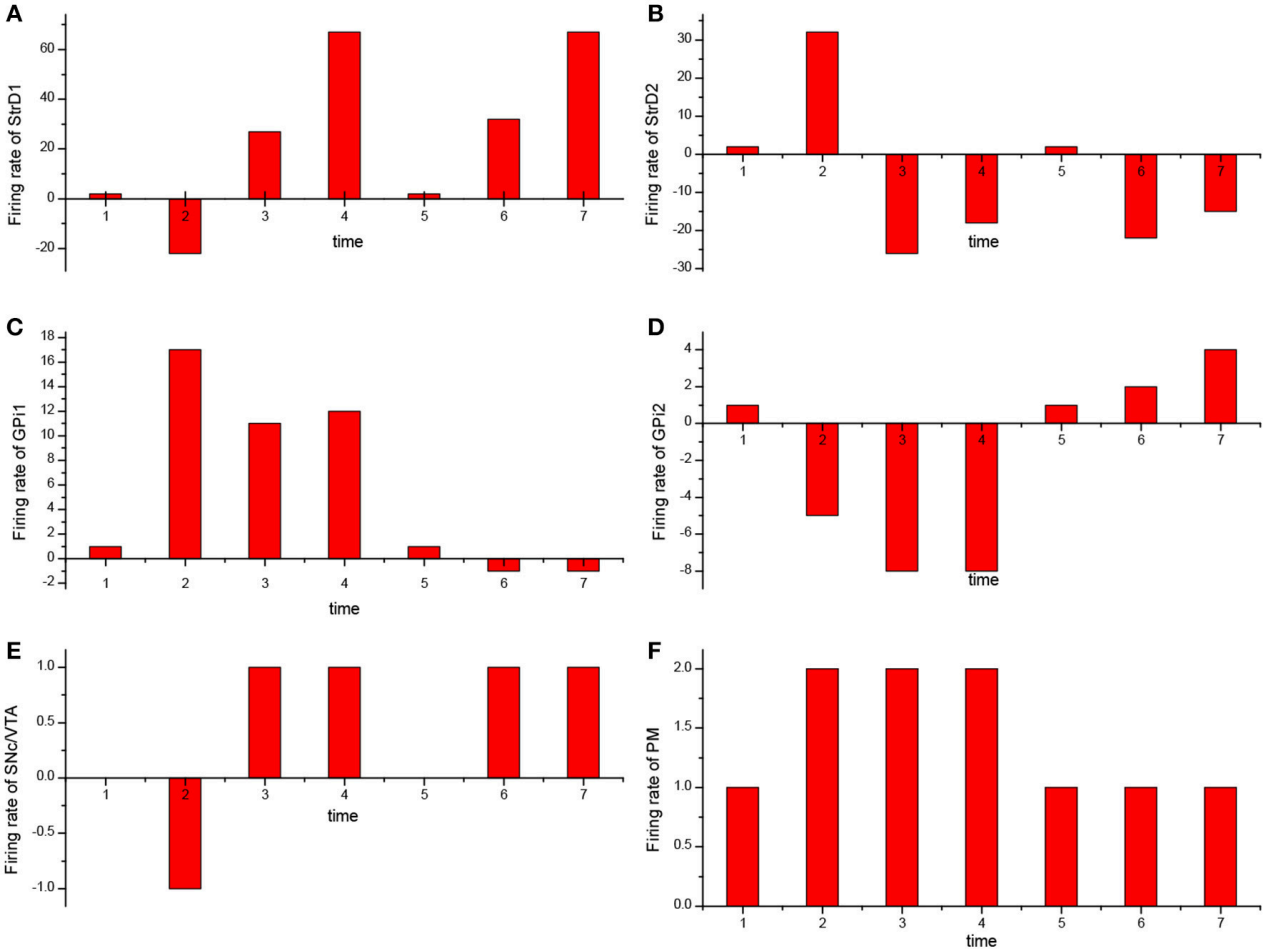
The change of firing rate for different brain areas [StrD1 **(A)**, StrD2 **(B)**, action 1 of GPi **(C)**, action 2 of Gpi **(D)**, SNc/VTA **(E)**, PM **(F)**] during the learning process. The X axis represents the time line of decision-making.

For the UAV obstacle avoidance task, the changes of firing rate of some main brain areas including StrD1, StrD2, SNc/VTA, Gpi, PM are depicted in Figure 10. Here, each firing rate is subtracted a baseline. The X axis represents the decision-making time line. At the first time, the UAV is situated in state 1. PM randomly chooses action 1 in Figure 10F. Then the action is incorrect and SNc/VTA carries negative reward in Figure 10E. It updates the connection weights between DLPFC and striatum. The weights between DLPFC and StrD1 are weakened, and the weights between DLPFC and StrD2 are strengthened. As a result, at the next time, the firing rate of StrD1 is lower (Figure 10A), while the firing rate of StrD2 is higher (Figure 10B). Then the firing rate of action 1 is higher (Figure 10C), while the firing rate of action 2 is lower (Figure 10D) in GPi. PM outputs action 2 (Figure 10F) due to the inhibitory effect of GPi. After choosing action 2, the level of SNc/VTA increases, and the StrD1 is strengthened while StrD2 is weakened. Thus, at the third time, the firing rate of StrD1 is higher, while the firing rate of StrD2 is lower, and PM continuously chooses action 2. When the state is changed to a new state, state 2 (at the fifth time), PM randomly chooses action 1. It is the correct action in state 2, thus SNc/VTA is higher, and the firing rate of StrD1 is higher while the firing rate of StrD2 is lower. Then PM continuously outputs action 1 to execute. These changes of firing rates in different brain areas support the effectiveness of the BDM-SNN model.

## 4. Discussion

This paper proposes a brain-inspired decision-making spiking neural network (BDM-SNN) model which is inspired by the decision-making circuit and mechanisms in human brain. It is more biologically explainable from three perspectives: (1) The model uses more brain-inspired SNN to simulate the connections and functions of brain areas. (2) The model combines DA regulation with STDP synaptic plasticity mechanism. (3) The model considers the effects of sub-areas in PFC and this model is relatively more comprehensive. To verify the effectiveness of the model, we apply it to the UAV autonomous decision-making tasks including the UAV flying through a window task and the UAV obstacle avoidance task. Experimental results show that the model can be easily applied to intelligent agent's decision-making tasks. We also detect the change of firing rate in different brain areas and the weights between some main brain areas. They are consistent with the prediction. These results show that the proposed BDM-SNN model could have a step forward toward human-like decision-making. The main contribution and novelty of this paper is that we propose a BDM-SNN model with more biological evidence, and we verify its applicability on the UAV autonomous decision-making tasks. Now we discuss the difference and similarity of our model with some brain-inspired models and reinforcement learning method.

Firstly, we take a brief review about TD learning algorithm (Sutton and Barto, 1998). TD learning algorithm uses experience to optimize strategy. At time *t*, agent is situated in state *S*_*t*_, and it chooses an action *A*_*t*_. Then the environment provides the next state *S*_*t* + 1_ and reward *R*_*t* + 1_ at time *t* + 1 as feedbacks. In TD learning, we estimate the value function of a state *V* (*S*_*t*_). *V* (*S*_*t*_) is used to estimate how good it is for an agent to be in a given state. Then the agent tries to optimize this *V* (*S*_*t*_) in order to achieve more reward. After executing an action, the *V* (*S*_*t*_) will be updated as Equation 14. Here δ_*t*_ is the TD error.

(14) $$\begin{array}{c} V\left(S_{t}\right)\leftarrow V\left(S_{t}\right)+\alpha \delta_{t}\\ \delta_{t}=R_{t+1}+\gamma V\left(S_{t+1}\right)-V\left(S_{t}\right) \end{array}$$

For the BDM-SNN model, DLPFC represents state and DA regulates the weights between DLPFC and striatum. So *W*_*DLPFC*−*StrD*1_ and *W*_*DLPFC*−*StrD*2_ are similar to the *V* (*S*) in TD learning algorithm. It has been proved that DA is responsible for carrying TD error (Hollerman and Schultz, 1998; Bayer and Glimcher, 2005). In the BDM-SNN model, the DA concentration is related to *r*_*end*_ as Equations 9 and 10 shown. Based on the definition of *r*_*end*_, we prove the similarity between *r*_*end*_ and δ_*t*_ as Equation 16 shown. Here, *R*_*b*_ (*S*_*t*_) approximately estimates *V* (*S*_*t*_) because *R*_*b*_ (*S*_*t*_) represents the basic value of state *S*_*t*_. The actual reward *r*_*end*_ is calculated by relative reward *r*_*t*+1_ − *r*_*t*_:

(15) $$\begin{array}{l} r_{end}=r_{t+1}-r_{t}\\ =\left[R_{b}\left(S_{t+1}\right)+\alpha Eva\left(S_{t+1}\right)\right]-\left[R_{b}\left(S_{t}\right)+\alpha Eva\left(S_{t}\right)\right]\\ =\alpha \left[Eva\left(S_{t+1}\right)-Eva\left(S_{t}\right)\right]+R_{b}\left(S_{t+1}\right)-R_{b}\left(S_{t}\right)\\ \approx R_{t+1}+V\left(S_{t+1}\right)-V\left(S_{t}\right)\\ \approx \delta_{t} \end{array}$$

The differences between the BDM-SNN model and TD learning are as follows:

**(1)**We simplify the DA concentration as Equations 9 and 10 shown. We optimize the weight updating mechanism in TD learning (added by the TD error) by Equations 11 and 12 (multiplied by DA concentration). The reason is that DA is a kind of neurotransmitter and the concentration is related to the degree of synaptic transmission.

**(2)**The BDM-SNN model can explain the TD learning method in reinforcement learning, and the model is more biological inspired. The SNN is not the only way to implement the decision-making but the more brain-inspired way to explore the effectiveness of human-like model.

In our previous work (Zhao et al., 2017), the proposed PFC-BG model mainly focuses on the PFC top-down biasing effect on BG. It is a mathematical optimization on Actor-Critic method with the inspiration of working memory and continuous DA regulation. The BDM-SNN model in this paper focuses on the simulation of brain decision-making circuit, which is a dynamic learning process with spiking neurons. Besides, on the UAV flying through a window task, the BDM-SNN model needs fewer steps than the PFC-BG model, as Figure 8 shown. In BDM-SNN model, the effects of MOFC and LOFC are taken into consideration. The coordination of DA and STDP strengthens the connections between MOFC and StrD1, and the connections between LOFC and StrD2. When agent receives positive reward, MOFC fires and facilitates the activity of StrD1. By this way, the activity of MOFC can facilitate the direct pathway. When agent receives negative reward, activity of LOFC facilitates the indirect pathway by strengthening the connections between LOFC and StrD2. To sum up, MOFC and LOFC can enlarge the difference between two competitive pathways and accelerate the decision-making process.

There are also other neurocomputational models inspired by decision-making mechanism in the brain. Frank et al. modeled the direct pathway and indirect pathway in brain decision-making with DA regulation in their model (Frank, 2005). They further took the effect of OFC into account (Frank and Claus, 2006). The main difference of network architecture between our method and Frank's works is the completeness of decision-making circuit. We model direct, indirect and hyperdirect pathways, while the hyperdirect pathway is ignored in Frank's works. Many decision-making models have not considered the STN brain area (Frank and Claus, 2006; Zeng et al., 2017) and the connection between DLPFC and thalamus (Gurney et al., 2015). The excitatory input from STN is essential for GPi for the other inputs (StrD1 and GPe) are inhibitory. Only the projection of STN can help GPe and GPi function. If there is no STN, GPe and GPi will never fire, and the thalamus can not obtain inhibitory input from BG. Then thalamus always outputs randomly action. The excitatory input from DLPFC is very important for thalamus due to the input from GPi is inhibitory. If there is no excitatory input from DLPFC, thalamus will never fire as well. To sum up, the excitatory input from STN and DLPFC is not only very necessary in human brain decision-making, but also computationally very important in our model.

Baston et al. considered DA and acetylcholine collective effect on the biologically inspired BG model (three pathway model). In this model, tonic activity of DA was also considered (Baston and Ursino, 2015b), while in our model, we only consider the phasic activity of DA (peak during positive feedback and dip during negative feedback). Baston et al. proposed a mathematical model to reproduce the main BG structures and pathways. This model contained both the dopaminergic and the cholinergic system to train synapses in the striatum (Baston and Ursino, 2015a). They further proposed a compartmental model of levodopa to build a general model of medicated Parkinson's disease (Baston et al., 2016). The main difference between our model and these works is that we take the effects of MOFC and LOFC into consideration.

**Figure 11:**
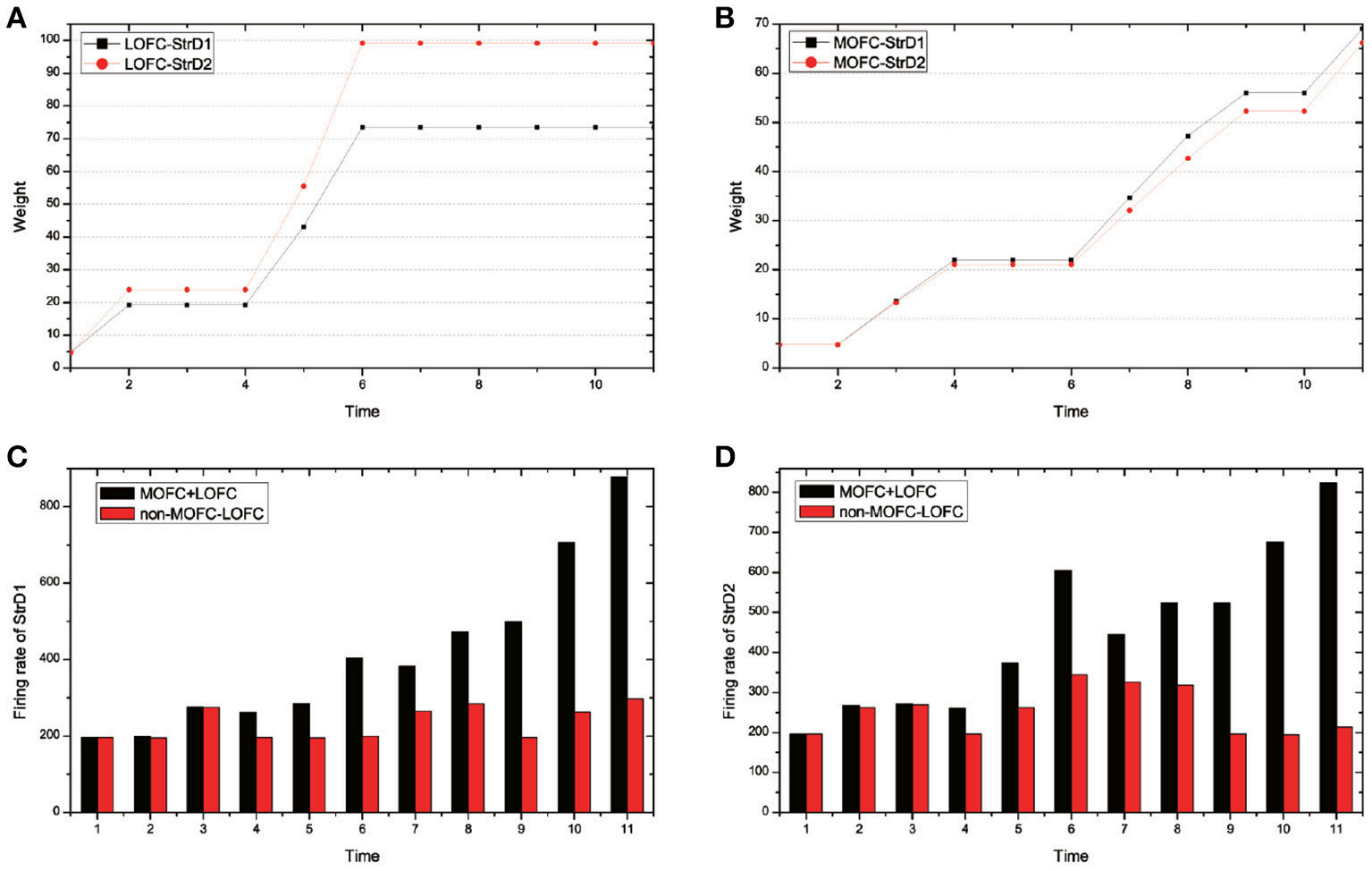
During the learning process, the change of weights between LOFC and StrD1, and LOFC and StrD2 **(A)**. The change of weights between MOFC and StrD1, and between MOFC and StrD2 **(B)**. When comparing the model with and without the effect of MOFC-LOFC, the change of firing rate of StrD1 **(C)** and StrD2 **(D)**. The red bars represent the firing rates without MOFC-LOFC, and the black bars represent the firing rates with MOFC and LOFC.

MOFC and LOFC are usually ignored in the existing works (Frank and Claus, 2006; Gurney et al., 2015; Zhao et al., 2017). For the UAV flying through a window task, Figure 11 shows the effect of MOFC and LOFC on accelerating decision-making. When obtaining positive reward, the MOFC-StrD1 connections will be strengthened. When obtaining negative reward, the LOFC-StrD2 connections will be strengthened based on STDP mechanism. Figure 11A shows the change of connection weights between LOFC and striatum. It is obvious that the weights between LOFC and StrD2 is larger than the weights between LOFC and StrD1. This conclusion is consistent with the prediction. Figure 11B shows the connection weights between MOFC and striatum. The conclusion is expected that the weights between MOFC and StrD1 is larger than the weights between MOFC and StrD2. Figures 11C,D compare the different firing rates of StrD1 and StrD2 in conditions with MOFC-LOFC and without MOFC-LOFC. By adding MOFC and LOFC brain areas, the firing rates of StrD1 and StrD2 are higher. By this way, the learning process is accelerated.

We test 100 steps and record the time cost of generating an action (the required iterative time). We compare the time cost with and without the effect of MOFC-LOFC. For the model with the effect of MOFC-LOFC, the average time cost is 51.423 steps, while for the model without the effect of MOFC-LOFC, the average time cost is 70.613 steps. This indicates that MOFC and LOFC can accelerate the decision-making process.

Although the current BDM-SNN model incorporates several important inspirations both from the connectome and the mechanisms perspectives from human brain, more inspirations can be used to further refine the model. For DA regulation mechanism, this paper focuses on the phasic burst of DA, which is triggered by unexpected rewards (Schultz et al., 1993). DA also exhibits tonic single spike activity, which refers to spontaneously occurring baseline spike activity (Grace and Bunney, 1984a,b; Grace and Onn, 1989). Baston et al. used the neurocomputational model to reproduce the function of BG, and analyzed the effects of different tonic dopamine levels on finger tapping task outcomes (Baston and Ursino, 2016). We will further work on the mechanism of tonic DA and integrate it with our current work. Besides, the detailed DA regulation mechanism needs to be further studied and added to the future work. In this paper, the state needs to be predefined before learning, an automatic state classification method during decision-making will be further investigated. Decision-making in human brain is a complex process, and it may contain more complex and subtle circuits and functions. We will further explore the multi-task decision making circuit and mechanisms in human brain, and optimize the existing model to conduct more complex tasks.

## Author contributions

FZ and YZ designed the study, performed the experiments and the analyses. YZ and BX were involved in problem definition, algorithm discussion, and result analysis. FZ and YZ wrote the paper.

### Conflict of interest statement

The authors declare that the research was conducted in the absence of any commercial or financial relationships that could be construed as a potential conflict of interest.
