# Corrigendum: A brain-inspired decision-making spiking neural network and its application in unmanned aerial vehicle

**DOI:** 10.3389/fnbot.2022.1092428

**Published:** 2022-11-29

**Authors:** Feifei Zhao, Yi Zeng, Bo Xu

**Affiliations:** ^1^Research Center for Brain-Inspired Intelligence, Institute of Automation, Chinese Academy of Sciences, Beijing, China; ^2^University of Chinese Academy of Sciences, Beijing, China; ^3^National Laboratory of Pattern Recognition, Institute of Automation, Chinese Academy of Sciences, Beijing, China; ^4^Center for Excellence in Brain Science and Intelligence Technology, Chinese Academy of Sciences, Shanghai, China

**Keywords:** spiking neural network, brain-inspired decision-making, dopamine regulation, multiple brain areas coordination, reinforcement learning, UAV autonomous learning

In the published article, there was an error. “Variable writing error in the algorithm 1.”

A correction has been made to **Materials and methods**, “*Experimental procedure*,” **Algorithm 1**. This sentence previously stated:

“DLPFC-DtrD1”

The corrected sentence appears below:

“DLPFC-StrD1”

The authors apologize for this error and state that this does not change the scientific conclusions of the article in any way. The original article has been updated.

## Publisher's note

All claims expressed in this article are solely those of the authors and do not necessarily represent those of their affiliated organizations, or those of the publisher, the editors and the reviewers. Any product that may be evaluated in this article, or claim that may be made by its manufacturer, is not guaranteed or endorsed by the publisher.

